# Modified all-suture fixation technique for anterior dislocation following conservative treatment of sternoclavicular joint injury: a case report

**DOI:** 10.3389/fsurg.2025.1732545

**Published:** 2026-01-09

**Authors:** Jiang-Long Wang, Zhi-Min Yuan, Jing-Rong Wen, Kang Li

**Affiliations:** 1 Department of Orthopaedics, Gannan Tibetan Autonomous Prefecture People’s Hospital, Hezuo, China; 2 Department of Orthopedics, Lanzhou University Second Hospital, Lanzhou, China

**Keywords:** all-suture fixation, case report, dislocation, sternoclavicular joint, surgery

## Abstract

Anterior dislocation of the sternoclavicular joint accompanied by articular disc avulsion following conservative treatment is a rare condition. A 58-year-old male suffered a shoulder acromioclavicular joint dislocation and a sternoclavicular joint injury without dislocation after being hit by a heavy object. Two weeks after the sternoclavicular joint was suspended, anterior dislocation occurred. He underwent a stable reduction using a modified all-suture fixation technique. At one-year follow-up, radiological assessment demonstrated maintained anatomical alignment and stable fixation, with complete restoration of daily activities and occupational function. The final Constant-Murley Score was 91, reflecting excellent functional recovery, and the patient reported high satisfaction. This case demonstrates that the modified all-suture fixation technique may offer reliable stabilization and favorable clinical outcomes in the treatment of anterior sternoclavicular dislocation associated with articular disc avulsion.

## Introduction

Sternoclavicular joint (SCJ) dislocation is a relatively rare injury, which accounts for 1%–3% of all upper extremity injuries (1). Anterior dislocation may result in persistent joint instability, which can impose functional limitations on patients with high levels of physical activity (2, 3). Anterior dislocation following conservative treatment of sternoclavicular joint injury is even rarer. This kind of non-acute anterior dislocation is rather difficult to handle, with few reported cases in the literature and no gold standard for treatment. Most cases adopt metallic fixation devices, and the subsequent surgery for internal fixation removal causes trouble for patients. In this report, we treated the patient with modified all-suture fixation and evaluated the treatment process and postoperative functional recovery of the patient.

## Case presentation

**Figure 1:**
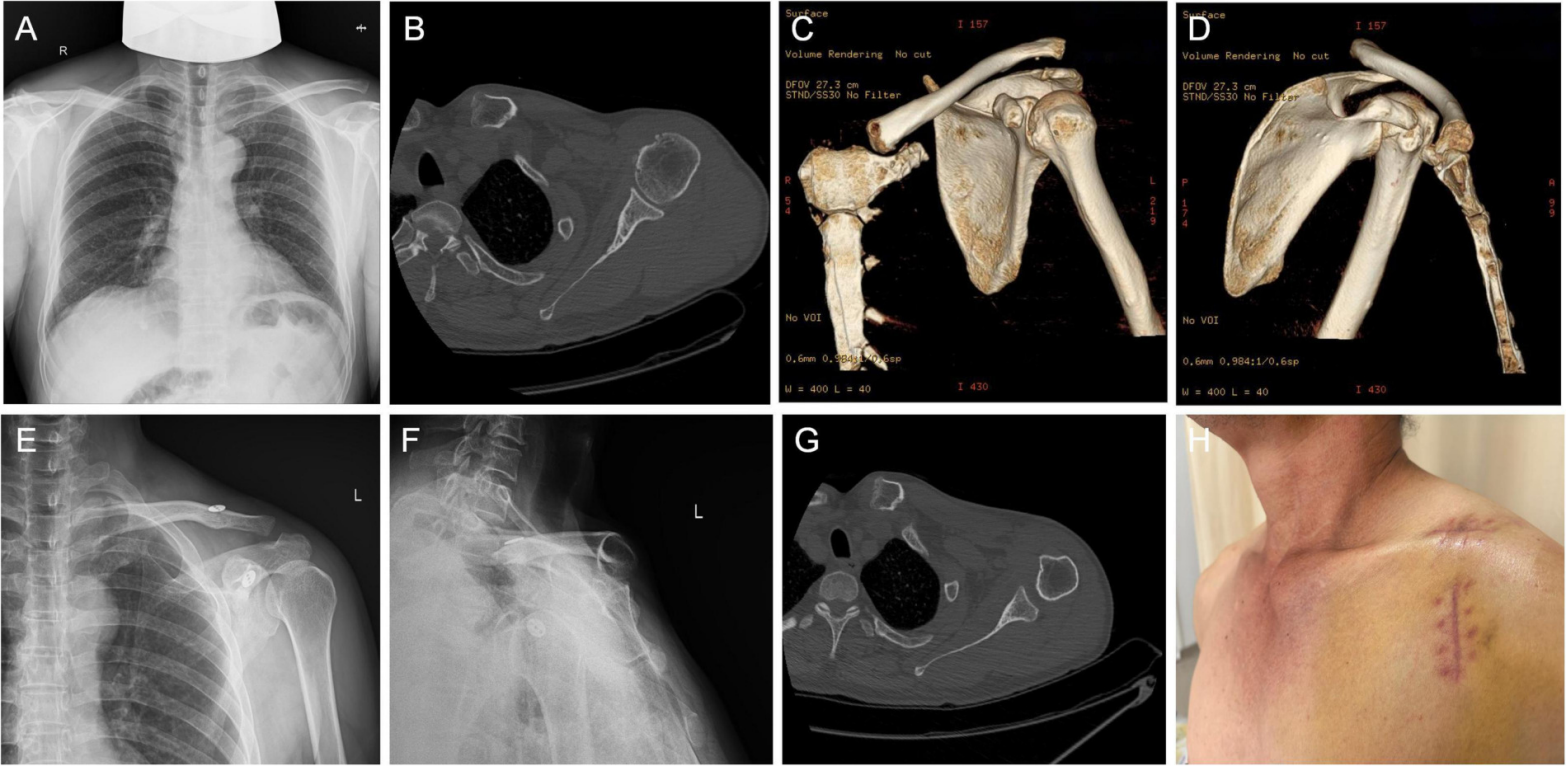
**(A–D)** Left ACJ dislocation, Rockwood type III, with no gross dislocation of the SCJ; **(E–H)** anterior dislocation of the left SCJ. Localized bulging at the left sternoclavicular junction.

A 58-year-old male patient was admitted to our hospital two weeks ago after being injured by a heavy object on his left shoulder while working at a construction site. He was diagnosed with a left ACJ dislocation, Rockwood type III, and underwent internal fixation with a Button plate. The left SCJ was injured but not dislocated, classified as Allman type I (4). He was advised to use a triangular sling for six weeks (Figures 1A–D). However, after two weeks of suspension, the patient removed the suspension device on his own and resumed daily activities. He then experienced local swelling at the sternoclavicular end, pain during movement, and increased limitations in daily life. He sought medical attention again. X-rays and CT scans showed a left anterior sternoclavicular joint dislocation (Figures 1E–H). Palpation revealed laxity at the medial end of the left clavicle, positive tenderness, and asymmetry of the sternoclavicular ends on both sides.

The patient underwent treatment for SCJ dislocation using the modified all-suture fixation technique. The surgical procedure was carried out under general anesthesia. The patient's head was gently tilted towards the right side. Antibiotics (cefuroxime sodium, 1.5 g) were intravenously infused 30 min prior to the surgical incision.

The prominent site of the SCJ dislocation was palpated. A transverse incision approximately 5 cm in length was made along the superior border of the medial clavicle. The subcutaneous tissue was dissected, and the muscular insertions on the superior and inferior aspects of the medial clavicle as well as the superior border of the manubrium of the clavicle were preserved. The rupture of the anterior sternoclavicular ligament and the joint capsule was visualized. Suture was used for elevation and marking to enable subsequent suture repair. After dissecting the anterior sternoclavicular ligament and joint capsule, it was observed that the medial end of the clavicle did not match the concave point of the manubrium sterni, the clavicle was displaced forward, and the articular disc was dislocated. Manual reduction was possible but could not be maintained. At a location 1.5 cm from the medial articular surface of the clavicle, drilling was carried out. A 2.0 mm drill bit was employed to create an oblique borehole from the anterior cortex of the clavicle towards the mid-point of the medial articular surface of the clavicle, forming one osseous tunnel superiorly and one inferiorly. Subsequently, at the site where the articular surface of the manubrium sterni matched that of the medial clavicle, drilling was initiated from a point 1 cm from the articular surface on the anterior cortex of the manubrium sterni. The drilling direction was oblique, aiming deeper into the articular surface of the manubrium sterni. Similarly, two 2.0 mm osseous tunnels were successfully established.

The non-absorbable ETHIBOND EXCEL (R) (Ethicon US, LLC) No. 5 suture was utilized. The suture was inserted through the four perforations in a parallel fashion. Specifically, one end of the suture was threaded from the superior aperture of the clavicle in a posteromedial direction and then passed through the superior aperture of the manubrium sterni in the reverse direction. Similarly, the other end of the suture was guided from the inferior aperture of the clavicle in a posteromedial direction and subsequently threaded through the inferior aperture of the manubrium sterni in the opposite direction. At this stage, the free ends of the suture were positioned anterior to the manubrium sterni, awaiting subsequent reduction before being pulled and knotted. To ensure the tensile strength of the suture, the firmness of fixation, and vertical stability, additional anterior-posterior drill holes were made at a distance of 0.5 cm from both the clavicle and the manubrium sterni. An identical suture was then passed through these holes to enhance vertical stability. After reduction of the articular disc, sutures are passed through the articular surface and the articular disc to maintain the stability of the articular disc.

**Figure 2:**
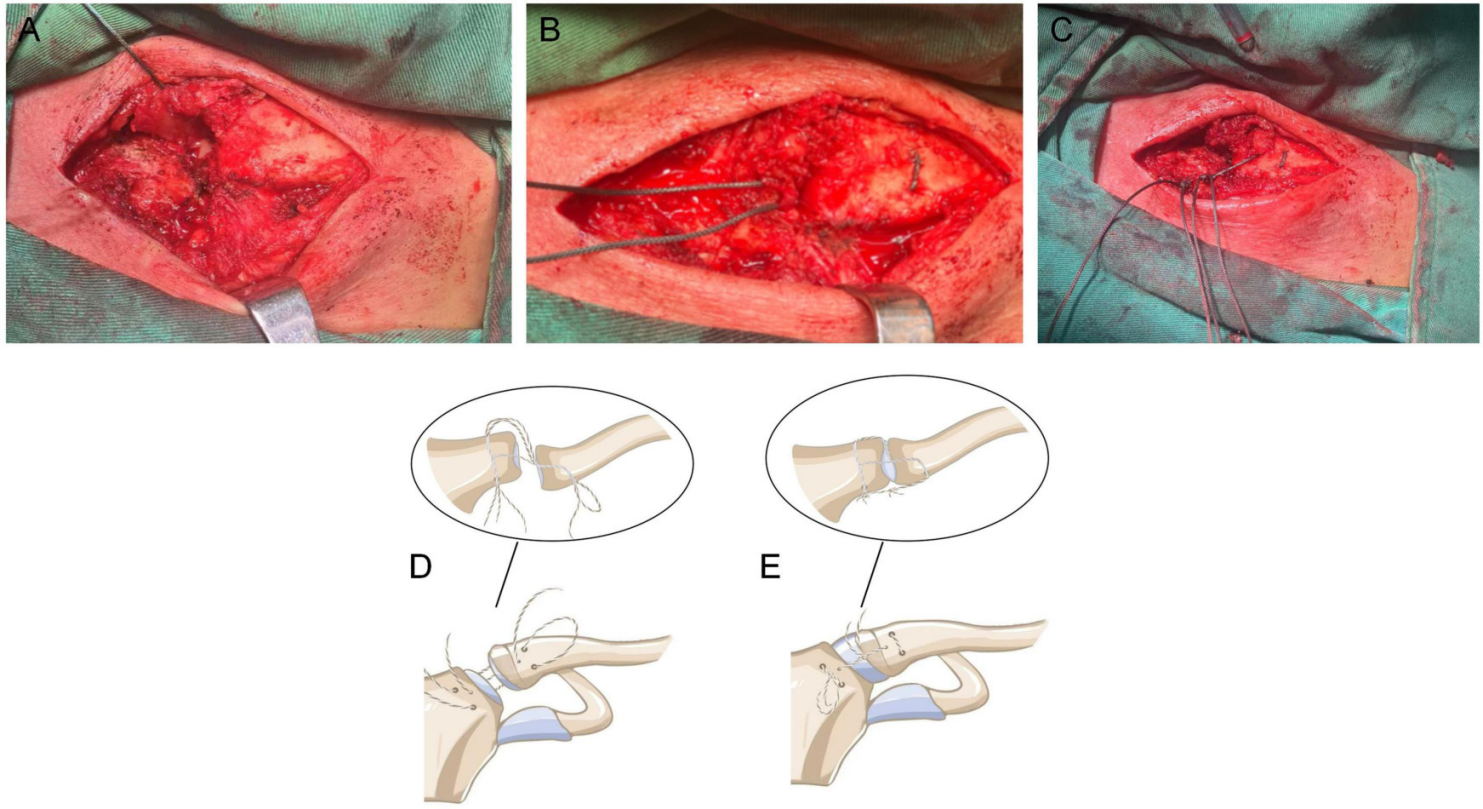
In **(A)**, avulsion of the articular disc is visible, and the suture was lifted for marking. In **(B)** the suture passed through the medial articular surface of the clavicle via an oblique bone tunnel and then through the articular disc. In **(C)** the two suture strands were fixed in parallel and perpendicular orientations. **(D,E)** Depict schematic diagrams of the surgical suture path.

Following manual reduction to achieve proper alignment of the sternoclavicular joint, anatomical reduction of the joint was visually confirmed under direct vision. With the assistant maintaining the reduced position, the sutures were knotted. Initially, the sutures arranged in parallel were ligated, followed by knotting of the vertical sutures. The remaining ends of the sutures were then used to repair the surrounding ruptured ligaments and joint capsule (Figures 2A–C).

The surgical site was thoroughly irrigated with normal saline. Subsequently, the incision was closed. A drainage rubber sheet was placed post-operation and removed 24 h later. The estimated blood loss during the surgery was approximately 50 mL. Subsequently, the patient continued to have the shoulder suspended with a triangular bandage for four weeks. During this period, active abduction and elevation of the shoulder joint beyond 90 degrees were avoided. Passive multidirectional shoulder mobilization under rehabilitation guidance was conducted during the four- to six-week period. Unrestricted active shoulder movement was gradually resumed after six weeks.

**Figure 3:**
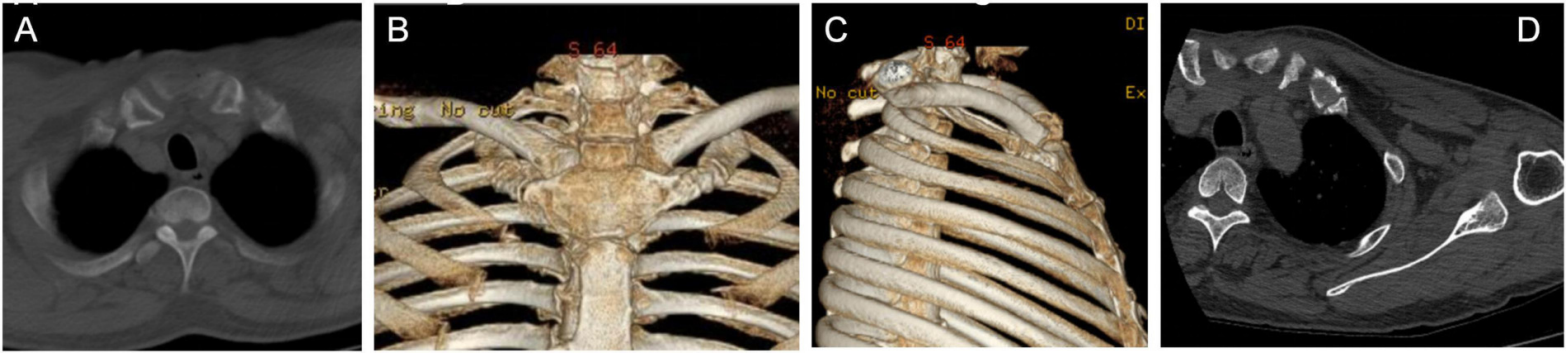
One month **(A–C)** and three month **(D)** postoperatively following surgery.

**Figure 4:**
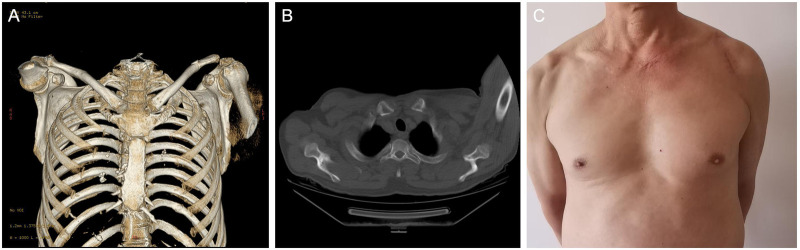
Six months **(A–C)** postoperatively following surgery. Visual inspection, as shown in the accompanying photograph, revealed symmetrical alignment of the bilateral SCJ.

**Figure 5:**
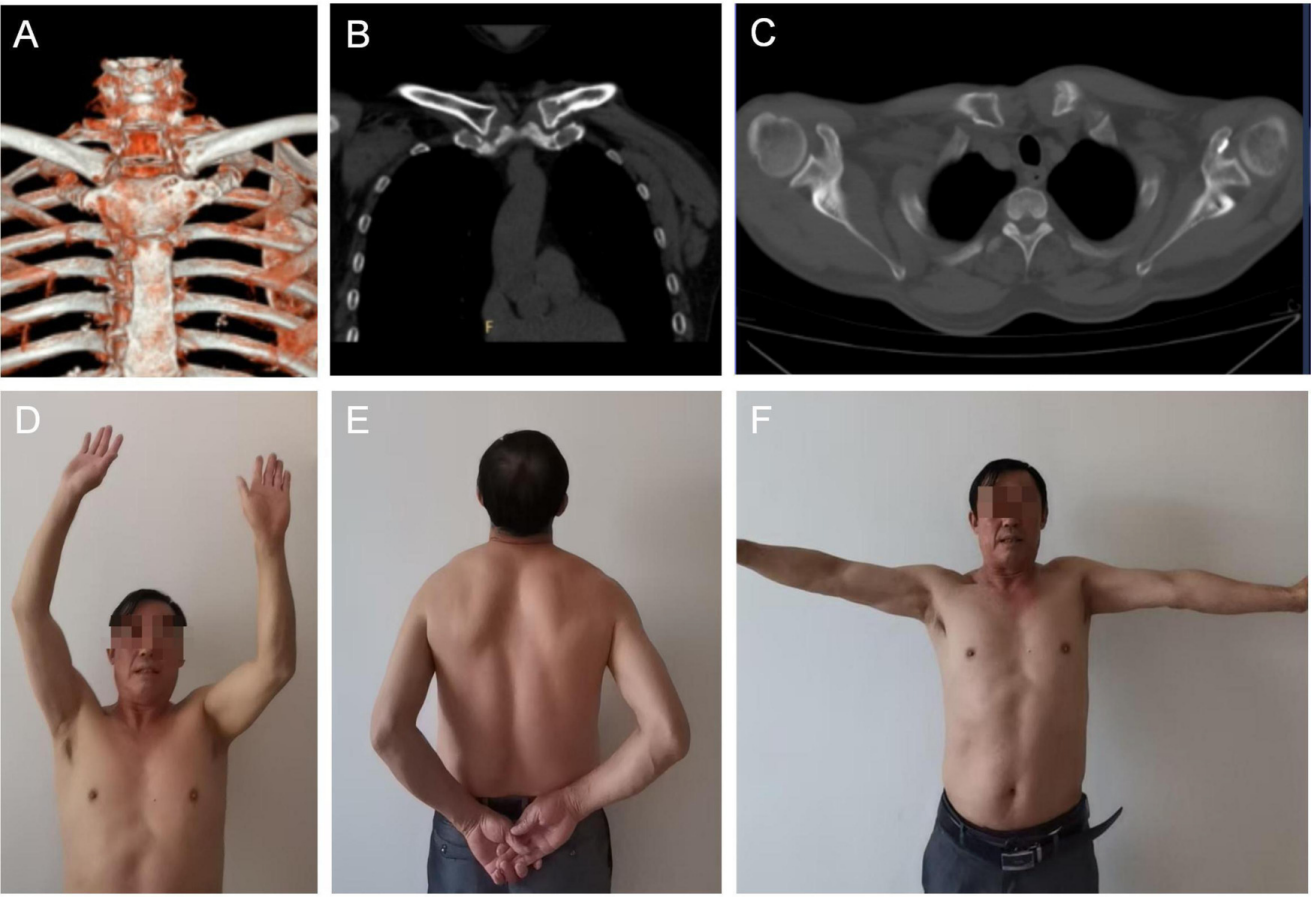
**(A–C)** show that the radiological examination one year after the operation indicates a good match of the sternoclavicular joint with no sign of dislocation. **(D–F)** show the functional status of the patient's shoulder joint in all directions one year after the operation.

Follow-up assessments were carried out at one month, three months, six months, and one year post-surgery. At the final follow-up (one year), the patient had fully restored normal shoulder movements and resumed daily life and work activities. Functional outcomes were measured using Constant-Murley scores (CMS) (5). The Constant-Murley Scores at three months, six months, and one year postoperatively were 71, 88, and 91 points, respectively (Figures 3–5).

## Discussion

The fibrocartilaginous clavicular portion plays a critical role in resisting compressive forces across the clavicular articular surface (6). Therefore, proper management of the articular disc is essential for maintaining joint integrity and function. Currently, for acute sternoclavicular joint dislocations, closed reduction followed by conservative management is regarded as the treatment of choice (1, 7, 8). However, in cases where conservative treatment fails, open surgery should be considered. The fixation techniques include the double-plate technique (9), Button Plate (10), Balser Plate (11), Hook Plate (12), and SCJ-specific Plate (13), allograft or autograft ligament reconstruction (14, 15), and all-suture techniques (16). There are notable discrepancies in the reported effects of various reconstruction techniques in the literature, this could potentially be attributed to factors such as the limited size of case series, variations in surgical indications, or differences in surgical techniques.

Among these, locking plates typically necessitate the insertion of screws at both the clavicular and sternal ends. Consequently, the most prominent issue is that the SCJ is rigidly fixed, potentially impeding the natural micromotion of the shoulder. The tip of the hook plate poses a risk of irritation and damage to the mediastinum behind the sternum. This is particularly true when the patient sustains another shoulder impact or other forms of violent trauma, during which the risk of mediastinal injury is significantly elevated. Similar concerns apply to the Balser Plate. All of the aforementioned metallic internal fixation devices are confronted with the issue of whether a second removal surgery is required, as well as the potential for fixation failure due to screw loosening in cases of osteoporosis.

The use of all-suture fixation for seven cases of anterior SCJ dislocation reported by Fandridis et al. (16) has drawn our attention. In their study, drill holes were created in the medial clavicle from anterior to posterior, and sutures were passed in parallel and subsequently ligated. Upon tightening, these sutures exerted a posterior and medial traction force on the medial clavicle, thereby stabilizing the sternoclavicular joint in its normal anatomical position. This technique represents a commendable biomechanical approach. However, based on our clinical experience, successful implementation requires extensive dissection of the soft tissues and periosteal attachments at the medial clavicle, resulting in considerable surgical trauma—an outcome inherently linked to the complex anatomical characteristics of this region. In males, the anterior—posterior thickness of the clavicle measures 19.3 ± 2.9 mm (17). Following anterior-to-posterior drilling, the passage of sutures typically necessitates dissection of the muscles attached to the medial clavicle. Furthermore, the extent of dissection and associated tissue trauma is substantial, rendering this phase of the procedure technically demanding. When there is a dislocation of the articular disc, it has no role in maintaining the repositioning of the articular disc.

Consequently, we introduced a modified technique in this case. Specifically, the drilling direction was changed from anterior-posterior to anterolateral-posteromedial. For the bony tunnel, the entry point on the clavicle is located on the anterior cortex, while the exit point is positioned at the midpoint of the medial articular surface of the clavicle. This orientation allows for complete visualization of the bony tunnel. The primary advantage of this approach is that it obviates the need for dissection of the muscles attached to the medial clavicle, thereby facilitating straightforward suture passage. For a more detailed illustration, please refer to Figures 2D,E.

Given that the patient had already undergone two weeks of shoulder immobilization following acromioclavicular joint dislocation surgery, to prevent interference with shoulder joint rehabilitation, we recommend a shortened period of immobilization—four weeks—after sternoclavicular joint dislocation surgery. Passive rehabilitation guidance should be initiated after this period to maximize recovery of shoulder function. At six weeks post-operation, unrestricted shoulder movements were gradually resumed. By three months post-surgery, the patient had resumed normal daily activities. At six months post-operation, the patient returned to work, although advised to avoid lifting heavy objects. At the one-year follow-up, full return to normal occupational activities was achieved. The patient reported satisfaction with the surgical outcome.

The advantages of our modified all-suture technique are as follows: First, the procedure is straightforward. Both ends of the bony tunnel in the clavicle are directly visible to the naked eye, greatly facilitating suture passage. Second, it ensures safety by avoiding potential irritation to the neurovascular structures posterior to the medial clavicle. Third, it minimizes surgical trauma, as dissection of the muscles surrounding the medial clavicle is not required. Fourth, two suture-passing techniques can be employed: parallel double-strand sutures or vertical single-site suture passage. These approaches provide multidirectional stabilization of the SCJ. Fifth, this approach is particularly applicable to cases with injury and displacement of the articular disc within the sternoclavicular joint.

Our study also has several limitations. First, the follow-up period was limited to one year, and it remains uncertain whether favorable clinical outcomes can be maintained in individuals engaged in heavy physical labor over the long term. Second, it is unclear whether this technique yields comparable results in patients with osteoporosis, a question that warrants further investigation. Third, additional biomechanical studies are needed to validate the stability and efficacy of this approach. Fourth, the current findings are based on a single case, and future multicase studies are warranted to validate and generalize these results.

## Conclusion

Anterior dislocation of the sternoclavicular joint accompanied by articular disc injury and displacement following conservative treatment is a rare occurrence, with only a limited number of cases reported in the literature. In this patient, modified all-suture fixation was performed, and follow-up assessments demonstrated stable maintenance of the reduced position and excellent functional recovery.

## Data Availability

The original contributions presented in the study are included in the article/Supplementary Material, further inquiries can be directed to the corresponding author.
